# Biology and structure of leukocyte β _2 _integrins and their role in inflammation

**DOI:** 10.12688/f1000research.9415.1

**Published:** 2016-10-04

**Authors:** M. Amin Arnaout

**Affiliations:** 1 Leukocyte Biology & Inflammation Program, Structural Biology Program, Nephrology, Center for Regenerative Medicine, Department of Medicine, Massachusetts General Hospital and Harvard Medical School, Boston, MA, USA

**Keywords:** inflammation, integrins, leukocytes, integrin structure

## Abstract

Integrins comprise a large family of αβ heterodimeric cell adhesion receptors that are expressed on all cells except red blood cells and that play essential roles in the regulation of cell growth and function. The leukocyte integrins, which include members of the β _1_, β _2_, β _3_, and β _7_ integrin family, are critical for innate and adaptive immune responses but also can contribute to many inflammatory and autoimmune diseases when dysregulated. This review focuses on the β _2_ integrins, the principal integrins expressed on leukocytes. We review their discovery and role in host defense, the structural basis for their ligand recognition and activation, and their potential as therapeutic targets.

## Introduction

**Figure 1.**
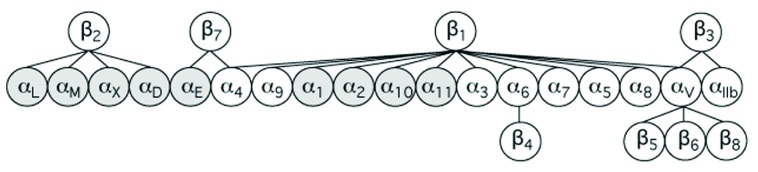
Mammalian integrins. This protein family consists of 24 α/β heterodimeric receptors assembled from 18 α-subunits and eight β-subunits. Nine α-subunits (shaded) contain an extra von Willebrand factor type A domain (αA or αI). The β _1_ integrins are the largest subfamily, with 12 known members.

Leukocytes circulate in the blood in a quiescent state before migrating into tissues to defend against invading pathogens or to participate in other immune functions. Improperly activated leukocytes can also be effectors of pathologic inflammation. Most leukocyte functions are dependent on members of the integrin family ( Figure 1). Leukocyte integrins comprise all four β _2_ integrins, the two β _7_ integrins α _4_β _7_ and α _E_β _7_, in addition to α _4_β _1_, α _5_β _1_, α _9_β _1_, and α _v_β _3_. Leukocyte integrins play key roles in the innate immune response, which include interaction of phagocytic cells with endothelium and the extracellular matrix, ingestion of complement-opsonized pathogens, degranulation, and cytokine production. They are also involved in lymphocyte proliferation, survival, and differentiation in adaptive immunity. Chemokines, cytokines, lipid signaling molecules, and “cross-talk” from other adhesion molecules regulate the functional state, density, and topography of leukocyte integrins. The leukocyte-specific β _2_ integrins are the most abundant leukocyte integrins and the first integrins to be studied functionally and structurally in these cells. In this review, we will focus on β _2_ integrins and their role in immunity and their structure and mechanism of their inside-out signaling. Many elements of the integrin outside-in signaling networks have been identified and were the subject of excellent reviews ^1– 4^ but are outside the scope of this concise review.

## Discovery of β _2_ integrins

The sequential steps leading to an inflammatory response were first documented by Julius Cohnheim in the frog’s tongue ^5^. He observed that local mechanical irritation induced first an increase in blood flow and then a slowing, at which time white blood cells began to roll and then halt, lining up the wall of venules, whereas red blood cells sped past them. Then some white blood cells began to creep across the wall into the extravascular space ^5^. Elie Metchnikoff discovered the phagocytic function of certain white blood cells by using the transparent avascular starfish larvae ^6^. His phagocytosis theory of inflammation complemented Paul Ehrlich’s humoral theory, which attributed bacterial killing to serum-derived “magic bullets”, identified soon after as antibodies and complement proteins. The identity of the molecules involved in leukocyte migration across venules and in phagocytosis remained unknown, however.

In 1979, an experiment of nature led us to the identification of the major surface receptors mediating leukocyte migration and phagocytosis (reviewed in 7). We investigated in a pediatric patient the basis for his life-threatening bacterial infections, impaired wound healing, persistent marked neutrophilia even during infection-free periods, but a paucity of neutrophils within infected tissues. His neutrophils failed to adhere to substrate, migrate across inflamed endothelium, or ingest serum-opsonized particles. We traced these phagocyte defects to a deficiency of a gp150 surface membrane glycoprotein complex ^8^. Monoclonal antibodies (mAbs) raised by us ^9^ and by others ^10– 15^ showed that the gp150 complex comprises four surface glycoproteins now known as CD11a (α _L_) ^16– 18^, CD11b (α _M_) ^19^, CD11c (α _X_) ^20^, and CD11d (α _D_) ^21^. Each CD11 glycoprotein non-covalently associates with a common 95 kDa glycoprotein (CD18, β _2_) ^13– 15, 18^ to form what is now known as the β _2_ integrin subfamily. Mutations in the CD18 subunit ^7, 22– 24^ resulted in its partial or complete failure to associate with the synthesized CD11 α-subunits, accounting for the variations in severity of the disease now known as leukocyte adhesion deficiency type I (LAD I) ^18, 25^.

## Tissue distribution of β _2_ integrins

β _2_ integrins are expressed only on leukocytes, but their expression varies among the leukocyte subpopulations. CD11a is expressed on all leukocytes but predominates on lymphocytes. CD11b predominates on myeloid cells, being the most abundant integrin on neutrophils, and is also expressed on natural killer (NK) cells, fibrocytes, and some mast cells, B cells, CD8 ^+^ T cells, and γδ T cells ^26– 33^. CD11c is most abundant on myeloid dendritic cells, predominating on macrophages and dendritic cells of the splenic white pulp and marginal zone and on pulmonary alveolar macrophages, and has a distribution similar to that of CD11b on NK, B, and T cells ^34^. CD11d is basally expressed on the majority of circulating human neutrophils and monocytes, on NK cells, and on a small fraction of circulating T cells ^35, 36^. In mice, CD11d expression is restricted to a small percentage of circulating leukocytes under basal conditions but predominates in splenic red pulp macrophages, lymph node medullary cord and sinus macrophages, and hemosiderin-containing bone marrow macrophages and is upregulated on phagocytes at local inflammatory sites ^35– 37^ and on differentiated macrophages, which may facilitate their retention at sites of inflammation ^38^.

## β _2_ integrin ligands

CD11a binds intercellular adhesion molecules (ICAMs) 1–5, telencephalin, endothelial cell-specific molecule-1 (ESM-1), and junctional adhesion molecule 1 (JAM1) ^39– 41^. CD11b is the most promiscuous β _2_ integrin; it has more than 40 reported ligands, including iC3b, ICAM1, 2, 3 and 4, fibrin(ogen), fibronectin, Factor X, Platelet Ibα, JAM-3, and some proteases (for example, proteinase 3) CD11c binds ICAM1, 4, iC3b, and vascular cell adhesion protein 1 (VCAM-1) ^42– 46^. Like CD11b, CD11c also binds heparin, various polysaccharides, and negative charges in denatured proteins ^26, 47– 49^. CD11d binds ICAM-3 and VCAM-1 ^50^ and, like CD11b, also binds several matrix proteins ^38^.

## Functional analysis of the individual β _2_ integrins

The defects in leukocyte adhesion demonstrated in patients with LAD I and in mice lacking CD18 ^51^ did not allow an assessment of the relative contribution of each of the four β _2_ integrins to the phenotypic abnormalities observed. Generation of mice deficient in the individual CD11 subunits revealed that knockout (KO) of CD11a (but not CD11b) in mice caused neutrophilia, which was not as severe as that found in CD18 KO mice, suggesting additional contributions by the other β _2_ integrins. No CD11a−, CD11b−, or CD11d KO mice developed the spontaneous infections observed in CD18 KO mice, suggesting that loss of all CD11/CD18 receptors is necessary to cause spontaneous bacterial infections. Homotypic aggregation and antigen-, mitogen-, and alloantigen-induced lymphoproliferation, which lead to defective host-versus-graft reaction and impaired tumor rejection, were reduced in CD11a ^−/−^ but not CD11b ^−/−^ or CD11c ^−/−^ leukocytes ^52, 53^. However, cytotoxic T-cell responses to systemic viral infections were normal in CD11a KO mice ^54, 55^, suggesting molecular redundancy or compensatory changes (or both) by other leukocyte integrins such as α4β1 or α9β1 ^56, 57^. This may explain the rarity of viral infections in patients with LAD I. Defective T-cell proliferation in response to the staphylococcal enterotoxin superantigen was more severe in splenocytes from CD18−, CD11b−, or CD11d KO mice than in CD11a ^−/−^ splenocytes but was normal in CD11c ^−/−^ splenocytes ^58^. The defects in CD11b ^−/−^ or CD11d ^−/−^ lymphocytes have been traced to transient expression of CD11b and CD11d on thymocytes, which appears to be required for normal T-cell development ^58^.

CD11a–d contributed in variable degrees to the adhesion of phagocytes to inflamed endothelium ^21, 42, 59, 60^. Transendothelial neutrophil migration in the tumor necrosis factor-induced air pouch inflammation model was reduced in CD11a KO ^61^, as in CD18 KO, but was surprisingly increased in CD11b KO mice ^60^. Migration within interstitial matrices was integrin independent ^62, 63^. Phagocytosis of serum-opsonized particles (with its associated oxygen free radical production, cytokine release, and degranulation) and phagocytosis-induced apoptosis in neutrophils were defective in CD11b ^−/−^ null mouse cells ^64^, confirming an essential role for CD11b in the programmed elimination of neutrophils that have already phagocytosed their target pathogens. Toll receptor-mediated responses were enhanced in CD11b ^−/−^ macrophages, rendering mice more susceptible to sepsis and endotoxin shock ^65^. Thus, whereas neutrophil adhesion to endothelium may require all four β _2_ integrins, transendothelial migration appears to be mainly CD11a dependent, while phagocytosis is mediated primarily by CD11b ^66^. Curiously, CD11b KO mice are obese ^67^, a phenotype not seen in patients with LAD I, suggesting a role for CD11b in regulating fat metabolism at least in mice. The number of mast cells in the peritoneal cavity is also reduced in CD11b KO mice ^27^, suggesting an additional role in mast cell development. Mast cells play an important role in the early peritoneal neutrophil response during experimental peritonitis in mice and this may explain the increased mortality of CD11b KO mice after acute septic peritonitis ^27^.

## Integrin structure

### The αA domain

**Figure 2.**
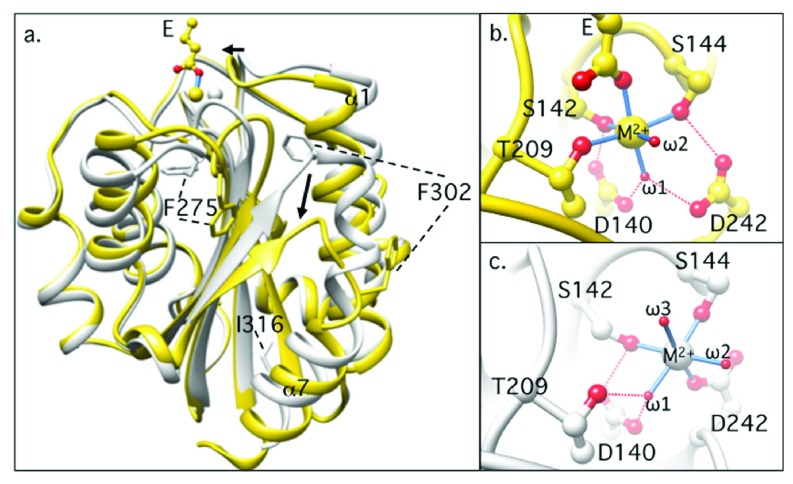
Structural comparisons of inactive and active αA domains. ( **a**) Ribbon diagrams showing the superposed structures of inactive (gray) and active (yellow) αA domain from the β _2_ integrin CD11b/CD18. Major conformational differences are indicated by arrows. The two phenylalanine residues (F275 and F302) buried in the inactive form are solvent exposed in the active state. A glutamate (E) from ligand is shown in the active (ligand-bound) state, ligating the metal-ion-dependent adhesion site (MIDAS) Mg ^2+^ monodentately. ( **b**, **c**) The MIDAS motif in the active ( **b**) and inactive ( **c**) states. The metal ion at MIDAS is coordinated by residues from three surface loops, and a carboxyl oxygen from ligand completes the octahedral coordinating sphere ( **b**). In the inactive state, an oxygen atom from a water molecule replaces the ligand oxygen, and D242 from the third surface loop moves in to coordinate the metal directly ( **c**). Coordinating oxygen atoms are in red, and hydrogen bonds are shown by dashed red lines. Direct bonds to the metal ion are shown as blue sticks. Water molecules are labeled ω1–ω3.

Structural studies of integrins began with the identification of a novel metal-ion-dependent adhesion site (MIDAS) in an extracellular von Willebrand factor type A (vWFA) domain (αA or αI domain) present in integrin CD11b ^68^. The vWFA domain is found in eight additional integrin α-subunits ( Figure 1) as well as in several structurally unrelated proteins ^69, 70^. αA from CD11b (CD11bA) mediates Mg ^2+^-dependent binding of the receptor to ligands ^68, 71^. αA also mediates ligand binding in the other αA-containing integrins. The first crystal structure of recombinant CD11bA showed a compact GTPase-like fold comprising a central, mostly parallel β-sheet surrounded on both sides by seven amphipathic α-helices ( Figure 2a). The catalytic site found at the apex in GTPases is replaced with MIDAS, where an Mg ^2+^ ion is coordinated by three surface loops ( Figure 2b). A solvent-exposed glutamate (E) or aspartate (D) from ligand completes an octahedral coordination sphere around the Mg ^2+^ ion ^69^. This crystal structure first explained why Mg ^2+^ is required for integrin binding to all physiologic ligands and why a solvent-accessible acidic residue from ligand is essential for binding to any integrin. Ligand-binding specificity in αA domains is imparted by the variable surface-exposed side chains surrounding the MIDAS motif.

The αA domain also exists in a second ligand-free “closed” conformation ^72, 73^, where the ligand coordinating carboxyl oxygen is replaced with a water molecule ( Figure 2c). Superposing the two structures shows the key tertiary changes associated with ligand binding: an inward movement of the N-terminal α1 helix, rearrangements of the metal-coordinating residues at MIDAS, and a 10 Å downward shift of the C-terminal α7 helix at the opposite pole to MIDAS ^72, 74^ ( Figure 2a). The key residues that stabilize the closed conformation have been identified, and mutations of some of these residues converted the closed into the open conformation ^75– 79^. Locking the open conformation with a pair of disulfides allowed crystallization of this form in the absence of ligand ^80, 81^. Crystal structures of αA domains from other integrins (for example, α _2_β _1_^82^), complement factors (for example, factors B and C2 ^83, 84^), certain matrix proteins ^85^, and microorganisms (for example, anthrax ^86^) were subsequently determined. These structures displayed the same basic conformational changes observed in CD11bA, underscoring their functional importance. In solution, recombinant wild-type CD11bA exists in an equilibrium where the proportion of the closed to the open state is nearly 9:1 ^75, 79^; the presence of ligand shifts this equilibrium in favor of the open state.

## The integrin ectodomain

**Figure 3.**
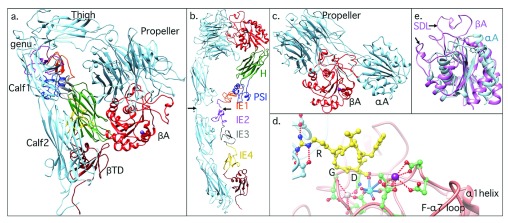
Structure of the integrin ectodomain. ( **a**) Ribbon drawing of the bent ectodomain from integrin α _V_β _3_. α _V_ is in light blue, and the 12 domains of β _3_ are shown in different colors for better visualization. The two tails would extend into the plasma membrane in the native integrin. ( **b**) Model of α _V_β _3_ ectodomain linearized by introducing breaks at the α and β genu (arrows). The modular nature of the ectodomain can be readily appreciated. H, hybrid domain; PSI, plexin-semaphorin-integrin. ( **c**) The integrin head from the αA-containing β _2_ integrin CD11c/CD18. ( **d**) Interactions between arginine-glycine-aspartate (RGD)-containing ligand peptide (yellow) and the α _V_β _3_ head. The peptide aspartate (D) completes the metal ion coordination sphere at metal-ion-dependent adhesion site (MIDAS), and ligand arginine forms salt bridges in the propeller pocket. αV and β _3_ residues are labeled light blue and orange, respectively. The three metal ions in β _3_ at MIDAS, adjacent to MIDAS (ADMIDAS), and ligand-associated metal binding site (LIMBS) are shown in cyan, magenta, and gray, respectively, and their coordinating residues displayed. The upper portion of the α1 helix and the loop between strand-F and α7 helix (F-α7) are also shown. Oxygen and nitrogen atoms are in red and blue, respectively. Hydrogen bonds and salt bridges (distance cutoff, 3.5 Å) are represented with dotted lines. ( **e**) Superposed structures of αA and βA domains. Shown are the two inserted loops in βA: the specificity determining loop (SDL) and heterodimer-association loop. The hydrophobic phenylalanine residue at the top of α7 helix that contacts α1 helix in αA is replaced in βA with an ionic interaction mediated by ADMIDAS ion.

The modular nature of an integrin was first revealed with the determination of the crystal structure of the ectodomain of the αA-lacking integrin α _v_β _3_ in its unliganded state ^87^ and when occupied by a cyclic peptide ligand containing the prototypical Arg-Gly-Asp motif ^88^. The α _v_ subunit is composed of a seven-bladed propeller domain, followed by a thigh domain and two large Ig-like Calf domains. The β _3_ subunit comprises an N-terminal plexin-semaphorin-integrin (PSI) domain, an Ig-like “hybrid” domain in which an αA-like domain (βA) is inserted, four successive epidermal growth factor (EGF)-like domains (IE1–4), and a novel membrane-proximal β-tail domain (βTD) ( Figure 3a, b). In the full-length integrin, Calf2 and βTD each is attached to a transmembrane (TM) domain and a short cytoplasmic tail. An unexpected feature of the α _v_β _3_ ectodomain is a sharp bending in the structure at the α-genu (between the thigh and calf1 domains) and the β-genu (within IE2) ( Figure 3a). Extension at the knees is expected to produce an extended integrin ( Figure 3b), which resembles the shape seen previously using rotary shadowing electron microscopy ^89^.

In αA-lacking integrins, the integrin head is formed of the βA and propeller domains ( Figure 3a, b), which associate non-covalently in a manner that resembles the association of the Gα and Gβ subunits of heterotrimeric G proteins ^87^. In αA-containing integrins, the head also contains the αA domain, which projects from a surface loop in the propeller ( Figure 3c). The heterodimer-disruptive point mutations found in the β _2_ (CD18) and β _3_ subunits in patients with LAD I and Glanzmann’s thrombasthenia (a bleeding disorder), respectively, map to the βA domain and commonly involve residues at the βA-propeller interface ^87^. As in αA domains, an acidic residue from ligand completes the octahedral coordination of Mg ^2+^ at MIDAS, an interaction stabilized by the arginine residue in the prototypical arginine-glycine-aspartate (RGD) motif, which inserts into a pocket in the propeller domain, making contacts with acidic residues in the pocket ( Figure 3d). Five metal ions (Ca ^2+^ or Mn ^2+^) occupy the bases of blades 4–7 of the α _v_ propeller and the α-genu ( Figure 3a, b); these may help rigidify the interfaces the thigh domain makes with the propeller base proximally and the top of Calf1 distally.

The structure of inactive βA is largely superimposable onto that of αA, except for two loop insertions: one forming the core of the interface with the α-subunit’s propeller and the second—the specificity determining loop, SDL—contributing to ligand binding as well as to the βA/propeller interface in some integrins (for example, α _IIb_β _3_) ( Figure 3e). In addition, a Ca ^2+^ ion at a site adjacent to MIDAS (ADMIDAS) in βA links the two activation-sensitive α1 and α7 helices, stabilizing this domain in the closed state; in αA, this ionic interaction is replaced by a hydrophobic one ( Figure 3e). In addition to the ADMIDAS ion, ligand-bound βA contains a ligand-associated metal binding site (LIMBS), which is occupied by Ca ^2+^ in ligand- or pseudoligand-bound integrins ^88, 90^. The structure of LIMBS in ligand-free integrins is regulated by the α-subunit’s propeller domain ^91^ and this may explain the variable metal ion occupancy of this site (sometimes also called synergy metal binding site).

**Figure 4.**
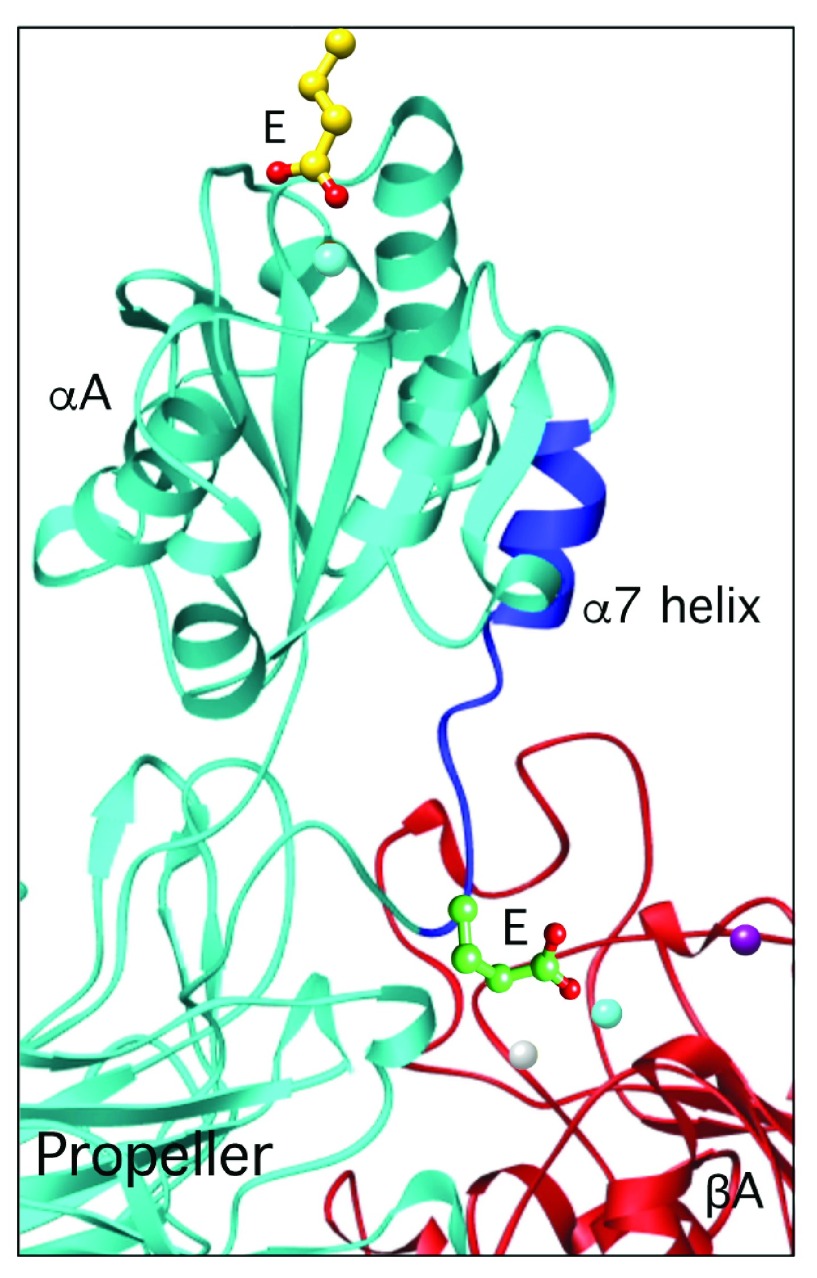
The ligand-relay model. The downward movement of the c-terminal α7 helix (dark blue) triggered by ligand binding to αA allows an invariant glutamate (E) at the bottom of the α7 helix to reach and ligate the βA metal-ion-dependent adhesion site (MIDAS) ion (cyan), thus relaying the ligand occupancy state of αA to βA.

In αA-containing integrins, the ligand-associated downward shift of the C-terminal α7 helix enables an invariant glutamate at the bottom of α7 to ligate the βA MIDAS ion ( Figure 4); mutation of this residue to alanine blocked integrin function ^92^. This led us to propose that αA serves as an intrinsic ligand for βA in αA-containing integrins. Blocking this coordination by the synthetic molecule XVA143 severs the αA link to βA and blocks integrin signaling ^93^. Support for this “ligand-relay” model came from the recent crystal structure of the CD11c/CD18 ectodomain ^94^. Thus, the βA domain transduces outside-in signals that are triggered by either extrinsic (in αA-lacking integrins) or intrinsic (in αA-containing integrins) ligands.

## Integrin transmembrane and cytoplasmic tails

The structure of the lipid-embedded α _IIb_ and β _3_ single-pass TM helices was determined by using solution nuclear magnetic resonance (NMR) spectroscopy ^95^. The structure revealed two dominant integrin TM association motifs or clasps: an outer (membrane-proximal) and an inner (membrane-distal) one that extends to include the adjacent cytoplasmic salt bridge between α _IIb_ and β _3_^96^. The two clasps maintain the integrin in the inactive state ^97^. Another structure in hydrophobic organic solvent invokes several differences in the membrane-proximal clasp regions, especially the helical conformation of α _IIb_ in the latter versus a reverse turn in the former structure ^98^. It is unclear at present whether this difference in the membrane proximal regions in the NMR structures reflects the nature of the lipid-like TM environment in which the TM domains were incorporated or reflects potential changes in response to binding of cytosolic regulators such as filamin ^99, 100^.

Binding of the N-terminal talin head to the membrane proximal NPxY/F motif in the β cytoplasmic tail destabilizes the α-β TM association ^101, 102^. Recruitment of talin to the plasma membrane requires ras-related protein 1 (Rap1) and its effector Rap1-GTP-interacting adaptor molecule (RIAM), and the latter is critical *in vivo* for inside-out signaling of β _2_ but not β _1_ or β _3_ integrins ^103, 104^. Kindlins have been reported to modulate receptor affinity ^105^ or avidity ^106^ or both. Kindlins bind the distal NPxY/F motif and a preceding threonine-containing region of the β cytoplasmic tail ^107^ but do not appear to destabilize α-β TM association ^108^. The structural basis for regulation of integrins by kindlins remains to be elucidated. Loss of kindlin 3 causes LAD III, a disease characterized by bleeding diathesis (defective α _IIb_β _3_ function) and defective leukocyte recruitment to sites of infection (defective β _2_ integrin function) ^105^.

## Integrin activation

Integrins are normally expressed in an inactive state on the cell surface. This is critical, as it allows leukocytes and platelets, for example, to freely circulate in blood with minimal aggregation or interaction with blood vessel walls. Binding of an agonist such as a chemokine or a cytokine (for example, granulocyte-macrophage colony-stimulating factor ^109^) to their respective receptors initiates inside-out signals that rapidly switch the integrin into the active state. Integrins stored in intracellular pools (for example, CD11b/CD18 ^18, 110, 111^ and α _IIb_β _3_^112^) are also recruited to the cell surface in response to agonists, but this process appears to follow the switch of the integrin to the active state ^113, 114^.

**Figure 5.**
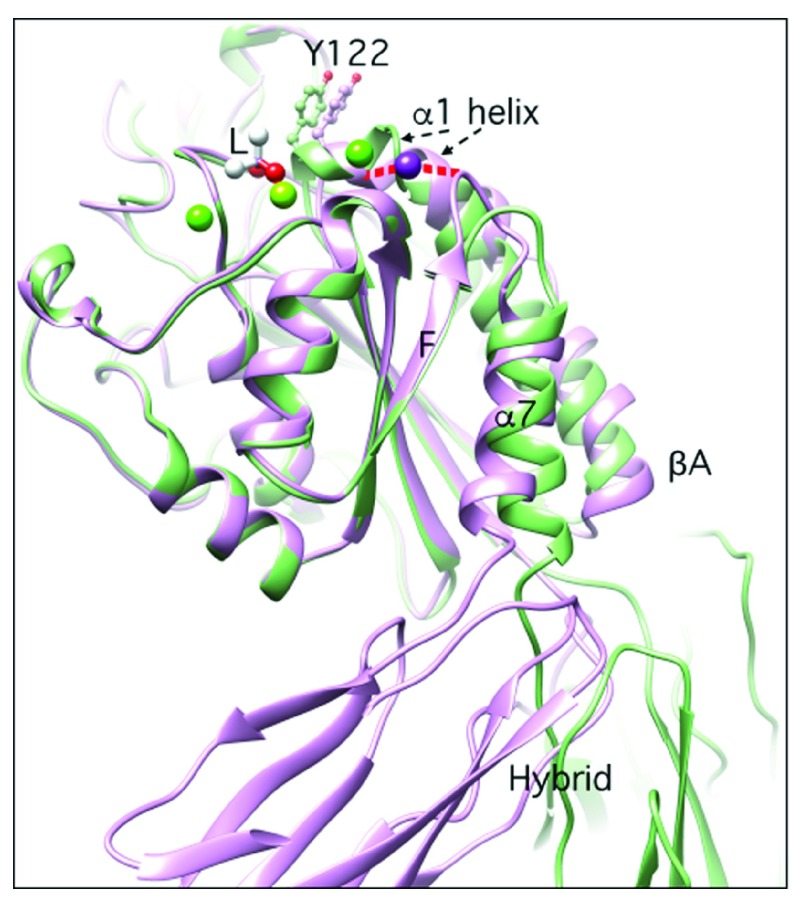
Structural changes in the βA domain following ligand binding. The superposed structures of βA domain of the β3 subunit in its unliganded (pdb 3ije) state and bound to cacodylate (acting as a pseudoligand, L) (pdb 1ty3) are shown in magenta and green, respectively. The main movements involve the α1 and α7 helices, loop F-α7, and the hybrid domain. In the unliganded state, helix α1 and Fα7 loop are connected via the adjacent to MIDAS (ADMIDAS) ion (magenta), and no metal-ion-dependent adhesion site (MIDAS) or ligand-associated metal binding site (LIMBS) atoms are detected. In the liganded state, a ligand oxygen coordinates MIDAS, and the α1 helix moves inwards (reported by tyrosine 122, Y122), bringing the ADMIDAS ion closer to the MIDAS ion and breaking the ionic contact with the F-α7 loop. These changes are coupled with a one-turn descent of the α7 helix and a 135° swingout of the hybrid domain in structures lacking the integrin leg domains.

The structural basis for integrin inside-out signaling is debated. Following publication of the bent ectodomain structure ^87^, a “switchblade” model envisioned that in the bent state, the ligand-binding site in βA (and αA in αA-containing integrin) is inaccessible to soluble ligand because of its proposed proximity to the plasma membrane. It is suggested, therefore, that the integrin linearizes to expose the ligand-binding site ^115^, which also allows an approximately 80° swingout of the hybrid domain and a switch of βA into high affinity ^90^ ( Figure 5). An alternate βTD-centric deadbolt model ^116^ proposed that the ligand-binding site in βA is already accessible to soluble macromolecular ligand in the native integrin ^117^ and can assume high affinity in the compact structure ^118^ and that genuextension occurs following binding of ligands or ligand-mimetic drugs to the cellular integrin ^119^. Movements of the membrane proximal βTD resulting from unpacking of the immediately distal TM segments disrupt βTD contacts with βA and hybrid domains, allowing the central switch of βA into the active state with minimal hybrid domain swingout ^118^.

Both models are supported by experimental data. Two-dimensional imaging using negative-stain electron microscopy (EM) showed a greater proportion of extended integrin ectodomains in the presence of the metal ion Mn ^2+^ (used as a mimic of inside-out signaling), and hydrodynamic studies showed an increase in the stokes radius of the α _V_β _3_ ectodomain in Mn ^2+^ ^115^. However, cryoelectron tomography showed that α _IIb_β _3_ maintained the compact (bent) conformation after Mn ^2+^ activation in a membrane environment ^120^. Differences in sample preparation, sampling bias in EM, and differences in ectodomain constructs may explain these discrepancies. A recent EM study of full-length integrin α _IIb_β _3_ in lipid-embedded nanodiscs showed a small increase in the extended conformation when the integrin was activated by talin ^121^. More recently, negative-stain EM of membrane-embedded full-length α _IIb_β _3_ showed that the active ligand-free α _IIb_β _3_ is mainly bent but that the ligand-bound receptor is predominantly extended ^122^. High-resolution quantitative dynamic footprinting microscopy combined with homogenous conformation-reporter binding assays showed that a substantial fraction of β _2_ integrins on the surface of human neutrophils assumed a high-affinity bent conformation ^123^. Because of the profound influence of the TM domains on integrin activation by inside-out signaling, settling the ongoing debate regarding the structural basis of integrin activation will likely require a three-dimensional crystal structure determination of a full-length native integrin in its native inactive and high-affinity states.

Ligand-bound integrins cluster, especially when occupied by multivalent ligands, and transduce outside-in signals leading to cell adhesion via new connections established between the integrin cytoplasmic tails and filamentous actin ^124^. In migrating cells, inward movement of the actin cytoskeleton from the site of assembly at the leading edge toward the cell center generates a pulling force across the nascent-integrin-matrix linkages and this unbends the liganded integrin and strengthens adhesion at these sites by accelerating recruitment of additional cytoskeletal and signaling proteins to the clustered integrins ^125^. As this pulling force increases in the moving cell, integrin-ligand bonds eventually break and integrins are endocytosed and this allows rear detachment and directional cell movement at the leading edge. Known adaptor proteins involved in integrin uptake and recycling have been recently reviewed ^126^.

## β _2_ integrins as therapeutic targets

Although β _2_ integrins are critical for innate and adaptive immunity, they can also induce serious pathology if improperly activated. Hyperadherent leukocytes may, for example, bind and injure the blood vessel wall, leukoaggregate intravascularly resulting in blocked capillaries or emboli, or compromise immune surveillance, thus contributing to inflammatory and autoimmune diseases. The finding that CD18 deficiency impaired the inflammatory response suggested that knockout of CD18 or CD11 or inhibiting their functions in leukocytes using antibodies may be beneficial in treating inflammatory or autoimmune diseases ^7^. A similar logic has been successful in targeting platelet α _IIb_β _3_ to inhibit pathologic thrombosis and this resulted in two orthosteric inhibitors, eptifibatide and tirofiban, and an allosteric inhibitor Abciximab, all three in clinical use ^127^.

Genetic deficiency of CD18, CD11a, or CD11b or targeting β _2_ integrins with various inhibitory antibodies in rodents ameliorated ischemia-reperfusion injury (IRI) in heart attacks, cerebral stroke, burns, and traumatic shock as well as autoimmune injury of the brain (multiple sclerosis), lung (asthma), and skin (psoriasis) and in native or transplanted kidneys (reviewed in 128). However, humanized forms of these mAbs failed when tested in patients with myocardial infarction, stroke, traumatic shock, multiple sclerosis, asthma, or acute rejection (reviewed in 128). An anti-CD11a mAb that showed promise in treating psoriasis was withdrawn because of fatal brain infections resulting from reactivation of JC virus ^129^. Inadequate design of some of the trials ^128^, important differences in immune responses between rodents and humans ^130^, and the relatively short follow-up period in the preclinical studies may have contributed to these failures. In addition, most clinical studies evaluating IRI syndromes used anti-CD18 antibodies, which might have acted allosterically to switch the integrin into the active proadhesive state. This scenario has precedence in β _3_ integrin-targeted mAb or small-molecule drugs, which act as partial agonists, unbending the integrin, thus exposing neoepitopes recognized by natural antibodies and leading to immune thrombocytopenia and bleeding, or inducing proadhesive outside-in signaling leading to paradoxical thrombosis ^131, 132^. Therefore, recent attempts have been made to solve the problem of partial agonism, making use of the advances made in structural biology of integrins. The central role of the A-domain in integrin activation and signaling made it a main focus of drug development efforts. The non-RGD-containing small molecules RUC-1, RUC-2, and UR-2922 were identified and act by inserting into the arginine-binding pocket in the propeller domain ^133, 134^, thus interfering with the stable binding of RGD-containing ligands. RUC-2 also binds to the β3 MIDAS residue E220 thus displacing the Mg ^2+^ at MIDAS ^133^. *In vivo* studies of RUC-1 administered intraperitoneally demonstrated anti-thrombotic effects in microvascular injury models in mice ^135^.

**Figure 6.**
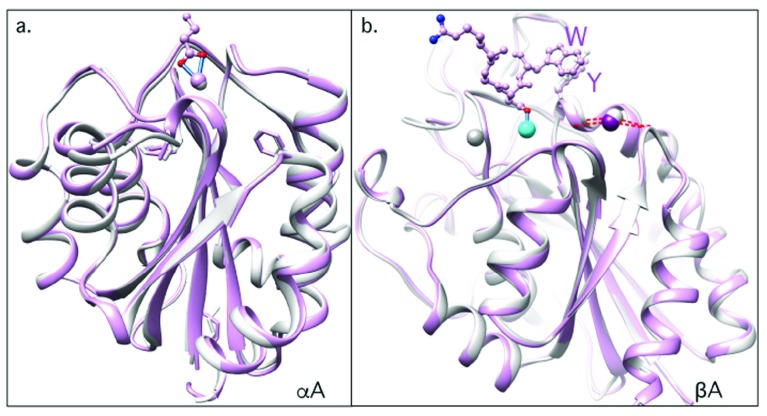
Structural basis of integrin inhibition by “pure” orthosteric inhibitors. ( **a**) Ribbon drawing showing structure of αA from the β _2_ integrin CD11b/CD18 bound to the pure ligand-mimetic antagonist mAb107 (in magenta). For clarity, only the ligand Asp of mAb107 is shown. The unusual symmetric bidentate ligation of the antibody-derived ligand Asp to a hepta-coordinated metal-ion-dependent adhesion site (MIDAS) Ca ^2+^ (blue sticks) prevents the tertiary changes associated with Mg ^2+^-dependent ligand binding. The superposed structure in gray is that of unliganded αA from CD11b/CD18. ( **b**) Structure of unliganded βA from α _V_β _3_ (pdb 3ije) (gray), superposed on the structure of βA in complex with a fibronectin-10-derived “pure antagonist” (magenta). Only the RGDW residues (in ball and stick) from ligand are shown (pdb 4mmz). Ligand-associated inward movement of the α1 helix and the resulting activating tertiary changes are prevented by a π–π interaction involving the ligand tryptophan (W) and βA’s tyrosine 122 (Y122). The ionic bridge (dashed red lines) between α1 and α7 helices is unaffected by binding of the pure orthosteric inhibitor. The metal ions at ADMIDAS, MIDAS, and LIMBS are in magenta (or gray), cyan, and dark gray, respectively.

We have approached the problem of partial agonism by identifying orthosteric inhibitors of integrin β _2_ (mAb107, ^117^) and β _3_ (a mutant high-affinity form of fibronectin-10, hFN10 ^136^) that do not induce the activating proadhesive changes in the αA or βA domains, respectively. mAb107 stabilized the inhibitory Ca ^2+^ in place of the proadhesive Mg ^2+^ at the CD11bA MIDAS, freezing the β _2_ integrin CD11b/CD18 in the inactive conformation ^117^ ( Figure 6a). hFN10 bound the βA MIDAS of integrin α _V_β _3_ and blocked the activating inward movement of the α1 helix ( Figure 6b), which is critical for integrin unbending and outside-in signaling ^136^. *In vivo* studies in monkeys showed that mAb107 ameliorated leukocyte-mediated inflammation in a severe IRI kidney model, salvaging kidney function from otherwise irreversible failure several months after a single injection of the mAb at the onset of IRI ^137^.

## Conclusions

Much has been learned since Cohnheim’s and Metchnikoff’s respective descriptions of leukocyte transendothelial migration and phagocytosis. The receptors involved have been identified, their critical role in innate and adaptive immunity defined, and their structures elucidated, revealing the atomic basis for their Mg ^2+^ dependency, ligand binding, and activation. Although putting the myriad interactions mediated by integrins into structural and biologic contexts remains a major challenge, the recent advances already made form a basis for structure-based discovery of effective and safer anti-inflammatory and anti-thrombosis therapeutics targeting these dynamic receptors.
